# Optimising flow without congestion using the venous‐arterial Doppler enhanced resuscitation framework

**DOI:** 10.1002/ajum.12388

**Published:** 2024-05-08

**Authors:** Jon‐Emile S Kenny, Philippe Rola

**Affiliations:** ^1^ Health Sciences North Research Institute Sudbury Ontario Canada; ^2^ Flosonics Medical Toronto Ontario Canada; ^3^ Division of Intensive Care Santa Cabrini Hospital Montreal Quebec Canada

**Keywords:** carotid corrected flow time, fluid responsiveness, fluid tolerance, Frank–Starling, venous Doppler, wearable technology

## Abstract

**Introduction:**

Ultrasonography as a guide for intravenous (IV) fluid therapy is increasingly accepted within the spheres of acute care. Initial investigations and protocols often focused on measures of arterial flow as an objective approach for personalising organ ‘perfusion.’ More recently, and with literature associating excessive IV fluid with adverse outcomes, venous ultrasound as a measure of organ ‘congestion’ is taking hold. Yet, arterial (i.e., ‘perfusion’) and venous (i.e., ‘congestion’) Doppler ultrasound measures are often performed separately and can be time‐consuming, especially for novices.

**Methods:**

We report a case, wherein venous and arterial Doppler were simultaneously measured using a wireless, wearable ultrasound as a means to optimise flow without congestion.

**Results:**

Before IV volume expansion, the patient had Doppler measures consistent with low central venous pressure (CVP) and stroke volume (SV). Following IV volume expansion, venous Doppler remained the same; however, carotid corrected flow time (ccFT) increased significantly.

**Conclusion:**

A framework for venous‐arterial Doppler enhanced resuscitation (VADER) can be used to guide IV volume in patients at risk for venous congestion.

## A case

A 67‐year‐old man was brought to the emergency room for confusion and general deterioration having been found on the floor of his home. Initial bloodwork showed renal dysfunction, elevated lactate, bilirubin, and hepatic enzymes. On examination, the patient had mild peripheral oedema. He was given crystalloid, and gastroenterology and critical care were consulted for possible hepatitis and sepsis, respectively. Point‐of‐care ultrasound (POCUS) done by the critical care team revealed marked venous congestion with a venous excess in ultrasound (VExUS) score^1^ of 3, normal lung ultrasound and severe biventricular dysfunction with poor right ventricular parameters.

He was diagnosed with severe splanchnic and cerebral congestion due to cardiomyopathy with predominant right‐sided failure and admitted to the ICU for diuresis. Furosemide 80 mg Q6H intravenous resulted in a net 4‐L negative balance in the first 24 h. A POCUS examination on day 6 revealed a VExUS score of 0, indicating the resolution of splanchnic congestion. While his vital signs were unchanged, his creatinine remained elevated and his mental status was poor. Thus, we were unsure as to whether the patient would improve forward flow with additional diuresis or, conversely, by giving preload.

## Our approach: Venous–arterial Doppler enhanced resuscitation framework

The therapeutic conundrum in this case is that of ‘optimising flow without congestion’. The patient initially received diuresis as he presented with congestive organ dysfunction – reflected by his high VExUS score.^1^, ^2^ A basic overview of the VExUS score is shown in Table 1.^1^

**Table 1:** Overview of venous excess ultrasound (VExUS) score.

VExUS zero	VExUS = 1	VExUS = 2	VExUS = 3
Inferior vena cava (IVC) less than 2 cm in diameter	IVC 2 cm or more in diameter with normal or mild venous Doppler abnormalities	IVC 2 cm or more in diameter with one severe venous Doppler morphology	IVC 2 cm or more in diameter with two or three severe venous Doppler abnormalities

Doppler patterns			
	Normal morphology	Mild abnormality	Severe abnormality
Hepatic vein Doppler	Systolic > diastolic wave	Systolic < diastolic wave	Completely retrograde systolic wave
Portal vein Doppler	Pulsatile fraction < 30%	Pulsatile fraction 30–49%	Pulsatile fraction of 50% or more
Intra‐renal vein Doppler	Continuous pattern	Discontinuous pattern with S and D waves	Absent S wave

S, systolic; D, diastolic.

With volume removal, his VExUS score normalised, yet days into his ICU stay, he maintained signs of organ hypoperfusion (i.e., elevated creatinine and confusion). Accordingly, uncertainty existed as to whether the patient would improve his forward flow with additional diuresis or, conversely, with preload.

**Figure 1:**
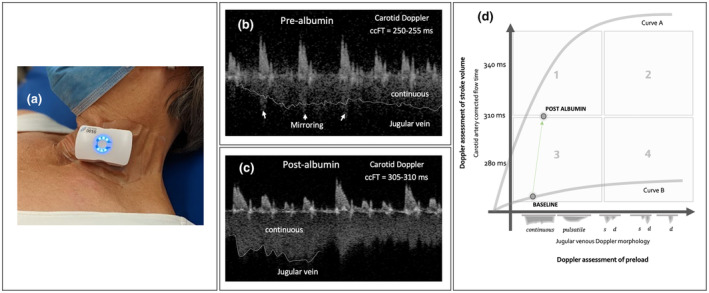
Data from the wearable device and haemodynamic framework. (a) The wearable Doppler ultrasound on a volunteer. (b) The carotid and jugular spectrograms from the patient prior to albumin. (c) The carotid and jugular spectrograms immediately following albumin. (d) The physiological framework discussed.

To help resolve the aforementioned, a wireless, wearable Doppler ultrasound (Figure 1a) was placed on the patient's neck, over the common carotid artery and internal jugular vein (IJV).^3^ This wearable ultrasound device is secured onto the skin with a dedicated adhesive and is composed of two continuous wave ultrasound transducers (i.e., transmit and receive); it connects wirelessly to a tablet for recording data. This wearable system has been deployed across a wide range of clinical interventions: for example, by volunteers undergoing simulated haemorrhage and transfusion, patients receiving intravenous fluid resuscitation, coronary artery bypass grafting in the operating room and during treatment of pericardial tamponade.^4^ The device automatically calculates and trends corrected flow time of the carotid artery (ccFT) over multiple cardiac cycles^5^; synchronously, the IJV Doppler spectrum is displayed. As this was not a formal investigation, there was no ethics review required for this case report from Santa Cabrini Hospital in Montreal, Quebec, Canada. Nevertheless, patient consent was obtained directly from the patient himself to publish non‐identifiable data.

As shown in Figure 1b, the patient had an irregular heart rhythm (i.e., atrial fibrillation) with a diminished ccFT (i.e., 255 ms over a 20 s average). Furthermore, his jugular Doppler suggested an ellipsoid or collapsed IJV. Given this, we surmised that the patient likely had a low stroke volume (i.e., low absolute ccFT) and low right atrial pressure (i.e., continuous jugular Doppler); his VExUS score at the time was zero.

We argue that integrating the venous and arterial data into a single conceptual framework is advanced by returning to first principles – the relationship between cardiac preload and stroke volume. We represent the Frank–Starling (or Sarnoff) relationship with venous Doppler on the *x*‐axis (as a surrogate for right atrial pressure) and the ccFT on the *y*‐axis (as a surrogate for stroke volume)^6^, ^7^, ^8^, ^9^ (Figure 1d). We fully acknowledge that neither of these Doppler measures are perfect proxies; nevertheless, this relationship integrates contrasting ultrasound surrogates into a single, physiological framework: one that is often used to explain cardiac physiology in the ICU. To further simplify the model, we dichotomise both axes. For jugular venous Doppler, we borrow from the work by Iida and colleagues as well as from our work with the VExUS score and consider the S > D wave pattern to dichotomise ‘low’ and ‘high’ venous filling when the patient is semi‐recumbent.^1^, ^10^, ^11^ For arterial Doppler, we note that a normal, resting ccFT in the semi‐Fowler position is approximately 310 ms (+/− 15–20 ms).^5^, ^12^, ^13^, ^14^ With these two thresholds, the relationship between the cardiac filling and output in Figure 1d is parsed into four quadrants, as elaborated below and in a recent review.^6^, ^9^, ^15^, ^16^

Quadrant 1, with low venous filling and high ccFT, might be considered ‘normal’ or ‘compensated’. A hypotensive or hypo‐perfused patient in this quadrant could have arterial vasodilation as a primary pathology. Quadrant 2 exhibits high venous filling with a normal ccFT. This phenotype suggests normal cardiac function with high venous return – as might be seen with pure volume overload (e.g., prior to dialysis).^13^ Rational therapy therefore implies volume removal, depending on context. Quadrant 3, into which our patient falls, intimates both low venous filling and stroke volume. Given the two extreme cardiac function curves (Figure 1d), quadrant 3 might represent a patient with *only* diminished venous return (e.g., volume loss and/or venodilation) with preserved cardiac function (curve A), for which rational therapy implies preload administration ± venous capacitance reduction (e.g., alpha agonists). However, quadrant 3 could also include a patient with *both* reduced venous return *and* cardiac function (curve B). This latter phenotype, termed *dynamic fluid intolerance*, is observed when a patient is found to be fluid intolerant only after a provocative manoeuvre such as a passive leg raise or mini fluid challenge.^6^ For this haemodynamic pattern, implied therapy is to improve cardiac function ± additional preload. Importantly, fluid loading a patient with *dynamic fluid intolerance* (i.e., quadrant 3, curve B) without improving cardiac function leads to quadrant 4 – high venous filling and low ccFT, as described in emergency department patients.^9^, ^16^ In quadrant 4, implied therapy is to improve cardiac function ± preload reduction. We emphasise that ‘improving’ cardiac function has multiple paths: rate and rhythm control, improving inotropy, reducing afterload, increasing afterload (e.g., dynamic outflow tract obstruction), relieving obstructive physiology (e.g., massive pulmonary embolus, dynamic hyperinflation and pericardial tamponade). Suffice to say that shifting the cardiac function curve up and leftwards is accomplished in various ways by targeting patient‐specific pathology.

Given our patient's continuous IJV Doppler morphology and relatively low ccFT, he was placed into quadrant 3. We followed continuous jugular venous and carotid arterial Doppler during and following 100 mL of 25% albumin; the patient maintained a continuous IJV Doppler pattern, but increased ccFT to 305–310 ms (Figure 1c). A + 40–50 ms ccFT augmentation is consistent with a ≥ +10% rise in stroke volume, as observed with both the wearable device and traditional, hand‐held approaches.^7^, ^12^ Thus, the patient moved from quadrant 3 upwards to quadrant 1 and we surmised that flow improved without congestion. Following albumin provision, the VExUS score remained zero, consistent with the continuous jugular morphology. The patient did not have concurrent stroke volume monitoring for example, left ventricular outflow tract (LVOT) velocity time integral (VTI) trending. However, change in the ccFT has compared favourably with bioreactance monitoring in undifferentiated shock,^17^ non‐invasive pulse contour analysis^12^ and ascending aortic VTI^18^ in volunteers undergoing severe, central hypovolaemia, intra‐pulmonary thermodilution in the critically ill^19^ and LVOT VTI measured by trans‐oesophageal echocardiography in post‐pump cardiac surgery patients.^20^ The patient's mental status and kidney function improved and he was transferred from the intensive care unit the following day.

## Conclusion

In conclusion, we underscore that precision resuscitation is nuanced. Congestion does not necessarily end where a low flow state begins; fluid intolerance^2^, ^6^ may not start with fluid unresponsiveness and these states can overlap.^15^, ^21^ Hence, the importance of finding an optimal haemodynamic position, or ‘sweet spot’, which may narrow with perturbed physiology. Haemodynamic frameworks and novel technologies may help ‘fine‐tune’ complex patients without invasive methods commonly limited to the ICU or the operator skill needed for ultrasound and cumbersome measures. The venous–arterial Doppler enhanced resuscitation framework could be used both to initially phenotype the shock state, as well as track resuscitation to ensure that interventions are pushing the patient's physiology in the desired direction, or at least not in an undesirable one.

## AUTHOR CONTRIBUTIONS

**Jon‐Emile S Kenny:** Conceptualization (equal); data curation (equal); formal analysis (equal); investigation (equal); methodology (equal); writing – original draft (equal). **Philippe Rola:** Conceptualization (equal); data curation (equal); formal analysis (equal); investigation (equal); methodology (equal); supervision (equal); writing – review and editing (equal).
